# Conceptual Validation of High-Precision Fish Feeding Behavior Recognition Using Semantic Segmentation and Real-Time Temporal Variance Analysis for Aquaculture

**DOI:** 10.3390/biomimetics9120730

**Published:** 2024-11-30

**Authors:** Han Kong, Junfeng Wu, Xuelan Liang, Yongzhi Xie, Boyu Qu, Hong Yu

**Affiliations:** 1 College of Information Engineering, Dalian Ocean University, Dalian 116023, China; konghan5219@gmail.com (H.K.); liangxuelan0315@gmail.com (X.L.); xyz1063042815@gmail.com (Y.X.); quboyu1208@gmail.com (B.Q.); yuhong@dlou.edu.cn (H.Y.); 2 Dalian Key Laboratory of Smart Fisheries, Dalian 116023, China; 3 Key Laboratory of Environment Controlled Aquaculture, Dalian Ocean University, Ministry of Education, Dalian 116023, China

**Keywords:** fish feeding behavior, semantic segmentation, deep learning

## Abstract

Aquaculture plays an important role in the global economy. However, unscientific feeding methods often lead to problems such as feed waste and water pollution. This study aims to address this issue by accurately recognizing fish feeding behaviors to provide automatic bait casting machines with scientific feeding strategies, thereby reducing farming costs. We propose a fish feeding behavior recognition method based on semantic segmentation, which overcomes the limitations of existing methods in dealing with complex backgrounds, water splash interference, fish target overlapping, and real-time performance. In this method, we first accurately segment fish targets in the images using a semantic segmentation model. Then, these segmented images are input into our proposed fish feeding behavior recognition model. By analyzing the aggregation characteristics during the feeding process, we can identify fish feeding behaviors. Experiments show that the proposed method has excellent robustness and real-time performance, and it performs well in the case of complex water background and occlusion of fish targets. We provide the aquaculture industry with an efficient and reliable method for recognizing fish feeding behavior, offering new scientific support for intelligent aquaculture and delivering powerful solutions to improve aquaculture management and production efficiency. Although the algorithm proposed in this study has shown good performance in fish feeding behavior recognition, it requires certain lighting conditions and fish density, which may affect its adaptability in different environments. Future research could explore integrating multimodal data, such as sound information, to assist in judgment, thereby enhancing the robustness of the model and promoting the development of intelligent aquaculture.

## 1. Introduction

According to the Food and Agriculture Organization of the United Nations, the aquaculture industry is growing rapidly, with a significant increase in the contribution of total global fish production [1]. In aquaculture, feeding costs account for 40% of the total costs. Therefore, a reasonable feeding strategy can save a significant amount of money for farmers. Traditional farming methods rely primarily on manual operations, which are usually inefficient. With the emergence of automatic bait casting machines, farmers began to use bait casting machines for feed feeding [2]. However, the current bait casting machine usually sets the feeding frequency and feeding amount according to the experience of the fish farmers, which lacks flexibility. Overfeeding of bait leads to feed waste and water pollution [3]. Underfeeding can lead to fish attacking each other for food [4]. Therefore, it is very important to adopt a reasonable bait feeding method to improve the feed utilization rate and prevent water pollution by residual bait.

Traditional fish feeding behavior recognition mainly relies on manual work. Farmers directly observe the activities of fish in the breeding pond to determine the feeding behavior. This method relies on the experience of farmers, who can judge the feeding status of fish by the aggregation of fish schools and the behavior of fighting for bait. However, manual observation has the disadvantages of strong subjectivity, high labor intensity, and short duration, so it is difficult to promote and use in large-scale fish farms [5].

With the progress in machine learning technology, fish feeding behavior recognition has begun to use data-driven methods. Algorithms such as Support Vector Machine (SVM) and Random Forest can improve recognition accuracy by learning the features and patterns in data. For example, researchers can use SVM classifier to classify the feeding behavior of fish, and let the algorithm automatically learn the feeding and non-feeding behavior characteristics of fish from a large amount of labeled training data.

In recent years, deep learning technology has gradually become the mainstream method for fish feeding behavior recognition due to its powerful feature learning ability. In particular, Convolutional Neural Network (CNN) features perform well, and can effectively capture the subtle movement patterns and interaction features in fish feeding behavior. For example, researchers can use 3D-CNNs to process video sequences to identify and classify the feeding behaviors of different fish.

Currently, fish feeding behavior recognition still faces significant challenges. First, the number of datasets related to fish is still limited, with no large public datasets available for fish feeding behavior. Second, some fish exhibit obvious feeding behaviors, and their rapid movements create splashes, which interfere with the recognition of feeding behaviors and reduce accuracy. This study identifies fish feeding behavior by analyzing the aggregation characteristics during feeding and the dispersal patterns after feeding, combining segmentation algorithms with a time-series model of fish feeding behavior. This approach effectively avoids misjudgments caused by fish occlusion, water splashes, and other interferences. The main contributions of this paper are as follows:

1. A fish feeding behavior recognition model based on semantic segmentation is proposed, which has good robustness and real-time performance. By combining the semantic segmentation model with our proposed FAIvar model, we can accurately distinguish the two states of fish feeding and non-feeding.
2. Introducing time series enables us to analyze fish states more accurately and in real-time. By examining the temporal evolution of fish behavior, we can capture dynamic changes in fish activity and thereby more accurately determine fish feeding behaviors.
3. We enhanced the Deeplabv3+ model by incorporating the Efficient Channel Attention (ECA) mechanism, significantly improving the segmentation accuracy of fish targets.
4. A voc format dataset (DLOUSegDataset) for fish segmentation is proposed, which contains 900 fish images.

## 2. Related Work

The high-precision identification of fish feeding behavior can provide effective bait casting methods for automatic bait casting machines, so as to reduce costs and increase production. Machine vision has been widely employed by some individuals in aquaculture because of its automatic, real-time, and non-contact characteristics [6]. Qiao employed machine vision technology to develop an image acquisition and processing system, through which they derived the feeding patterns of fish schools and feeding strategies based on image processing results [7]. Atoum developed an automatic feeding system for high-density aquaculture ponds based on SVM (Support Vector Machine) classifier. Traditional machine learning relies heavily on hand-extracted features, which leads to low accuracy and low robustness in the recognition of fish feeding behavior [8]. Zhou obtained binary images of fish by using image enhancement methods and calculated the grouping index of fish feeding behavior using Delaunary triangles [9]. However, this method is difficult to apply when the fish are occluded. With its powerful feature learning ability and higher accuracy, deep learning has quickly become widely used in fish target detection [10,11], fish length and weight measurement [12,13], fish individual recognition [14,15], fish disease recognition [16], fish feeding behavior recognition [17], and other aspects [18]. Among them, fish feeding behavior recognition can directly save costs for farmers. Zhu used the lightweight network MobileNetV3-Small to classify the feeding status of perch, and the accuracy was improved compared with the traditional machine learning method [19]. Zhang used convolutional neural network to classify Atlantic salmon into two states: feeding and non-feeding [20].

Fish feeding is a continuous and rapid process. Compared with 2D convolution, using 3D convolution for behavior recognition can better extract its features in the time dimension [21] using combined spatial and temporal information through spatial grids, 3D convolutions, and LSTM recurrent networks. This method can automatically capture the behavior of salmon during swimming. Ubina input optical flow frames into 3D convolution for fish feeding behavior assessment and obtained good results and improved the real-time performance of the model to a certain extent. Although 3D convolution can be used to distinguish fish feeding behavior, this method consumes a lot of time and struggles to perform real-time behavior recognition [22]. Table 1 provides a comprehensive overview of fish behavioral recognition, summarizing key methods, approaches, and their corresponding characteristics.

Considering the excellent performance of Deeplabv3+ in semantic segmentation, this paper selects Deeplabv3+ as the basic model [24]. Firstly, the semantic segmentation model is used to segment the fish target, and the segmentation results are input into the FAIvar model. Compared with the fish feeding behavior recognition method mentioned before, this method is not only fast and robust but also takes into account time dimension information. The use of a segmentation method can avoid phenomena such as splash reflection caused by fish in the process of feeding behavior, making the recognition results more accurate.

## 3. Methodology

### 3.1. OverAll

Fish feeding behavior recognition plays a crucial role in the aquaculture industry. The accurate identification of fish feeding behavior can achieve accurate feeding, improve the feed utilization rate, reduce costs, and effectively protect the water environment. Therefore, this study aims to explore a fish feeding behavior recognition model based on semantic segmentation, which combines machine vision and deep learning technology to achieve the real-time and accurate recognition of fish feeding behavior.

Our research method is based on semantic segmentation technology and aims to recognize the feeding behavior of fish. We used the Deeplabv3+ model, which has excellent image segmentation ability and can accurately segment fish from an image. Deeplabv3+ leverages the Atrous Spatial Pyramid Pooling (ASPP) mechanism to capture multi-scale contextual information, making it highly effective for detecting feeding behaviors where fish appear in varying sizes and positions within the frame. Its encoder–decoder architecture further enhances the segmentation of object boundaries, which is particularly advantageous in high-density aquaculture environments with overlapping or clustered fish. Moreover, Deeplabv3+ strikes an excellent balance between segmentation accuracy and computational efficiency, making it well-suited for real-time applications, including deployment on edge devices such as the NVIDIA Jetson Nano. On this basis, we divided the image into four regions of equal size and calculated the maximum area percentage and the fish area percentage in each region, respectively. We observed that when the baits are fed, the fish rapidly gather into one of the regions, resulting in significant changes in the maximum area percentage of the region and the percentage of fish area in each region. By monitoring these changes, we can accurately judge the feeding behavior of fish, which provides an important reference for aquaculture management and ecological environment monitoring. Our method not only combines advanced computer vision technology but also fully considers the understanding of fish behavior. It provides an efficient and accurate solution for real-time monitoring and analysis of fish feeding. In addition, due to its flexibility, our method can adapt to the monitoring requirements of different environments, providing valuable data support for related research and applications. In summary, our study provides an innovative method for the automatic recognition of fish feeding behavior, which has broad application prospects and scientific research significance.

**Figure 1:**
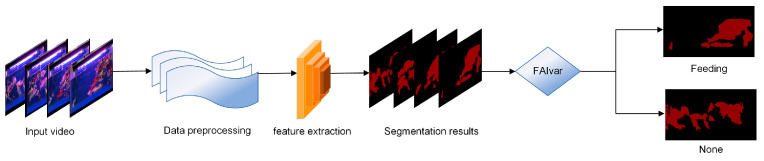
Flowchart of FAIECA-Deeplabv3+ for fish feeding behavior recognition.

In addition, we further optimized the Deeplabv3+ model by combining it with the ECA mechanism [25]. This improvement not only improves the segmentation accuracy of fish targets but also enhances the model’s perception of key fish features. By introducing the ECA mechanism, our improved model can capture the key information in the image more accurately and then improve the performance and stability of fish semantic segmentation, which provides more powerful support for practical applications. Figure 1 shows the flow chart of the fish feeding behavior recognition model method in this paper.

### 3.2. Semantic Segmentation Module

The earliest semantic segmentation methods can be traced back to traditional methods based on image segmentation and feature extraction. These methods usually rely on hand-designed features and classifiers, such as edge detection, color segmentation, etc. For example, both Graph Cut-based methods and edge detection-based methods are early semantic segmentation techniques, but they often struggle to deal with complex scenes and semantic categories.

With the rise in deep learning, especially the development of convolutional neural networks (CNNs), semantic segmentation has entered a new stage [26]. Fully Convolutional Network (FCN) is the first model to successfully apply deep learning for semantic segmentation, which replaces the fully connected layer of the traditional convolutional neural network with a convolutional layer to achieve end-to-end pixel-level classification [27]. The emergence of the FCN model marks the transition from traditional methods to deep learning methods for semantic segmentation.

Subsequently, a series of optimization models for semantic segmentation tasks have emerged. U-Net is a widely used semantic segmentation model [28]. By combining an encoder and a decoder, it can obtain global information and local information at the same time, which improves the accuracy and robustness of segmentation. SegNet is another commonly used semantic segmentation model [29]. It uses the index of the pooling layer for upsampling, which reduces the number of model parameters and calculation, and improves the efficiency and speed of the model.

**Figure 2:**
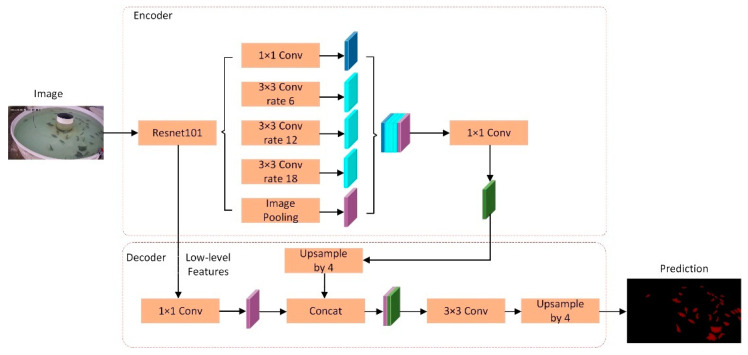
Flowchart of DeepLabv3+.

DeepLab series is a group of advanced semantic segmentation models. It adopts techniques such as atrous convolution and spatial pyramid pooling, which can effectively capture semantic information at different scales and improve the accuracy and generalization ability of segmentation. DeepLabv3+ is an advanced semantic segmentation model that inherits the advantages of deep learning models and makes a series of innovations on this basis, bringing significant improvements to semantic segmentation tasks. The model uses dilated convolution to expand the receptive field so that it can effectively capture semantic information at different scales and improve the model’s ability to understand image details and contexts. At the same time, DeepLabv3+ uses a multi-scale feature fusion module to effectively fuse different levels of semantic features, thus further improving the accuracy and robustness of semantic segmentation. In addition, the introduced spatial pyramid pooling module makes it possible to extract features and expand receptive fields at different scales, which enhances the expression ability of the model for image semantic information. In summary, the DeepLabv3+ model has excellent performance and efficiency in semantic segmentation tasks, and is suitable for a variety of complex scenarios and application requirements. The structure diagram of DeepLabv3+ is shown in Figure 2.

### 3.3. ECA Mechanism

Attention mechanism is a technique that imitates the way the human visual system works and is used to strengthen a neural network to pay attention to important parts of the input data. It allows a model to dynamically adjust its attention in order to focus on the most meaningful information for the task at hand when processing the input data. Segmentation tasks typically involve dividing the input data into multiple different parts or regions and classifying or labeling each part. Due to the complexity and diversity of input data, the importance of different regions may vary between them. In this case, the attention mechanism can help the model focus on the regions that are most important for the task at hand, thereby improving the accuracy and efficiency of segmentation.

**Figure 3:**
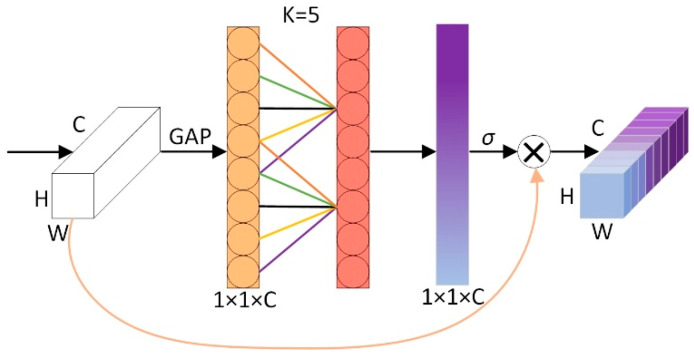
Structure diagram of ECA mechanism.

The ECA mechanism is an efficient neural network module, and its structural diagram is shown in Figure 3. It is designed to effectively capture channel relationships in images and enhance feature representations through the channel attention mechanism. Firstly, global average pooling is used for each channel separately to extract the global information of each channel. Then, a one-dimensional convolution calculation is performed on each channel and its k adjacent channels to realize the local cross-channel information interaction. This design enables the module to effectively capture the dependencies between long-distance channels while maintaining high efficiency, thus improving the performance of the model.

**Figure 4:**
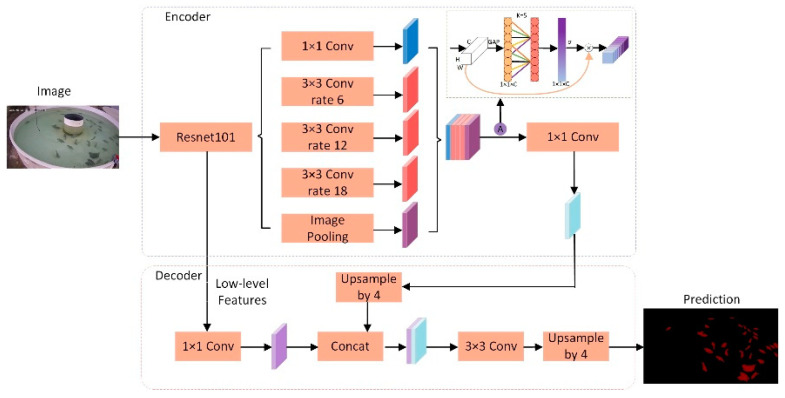
Flowchart of DeepLabv3+ with added ECA mechanism.

In the fish segmentation task, the ECA mechanism applied in the DeepLabV3+ model has significant benefits. By introducing the ECA mechanism, the model can better understand the relationship between different regions in the input image, thereby improving the accuracy and robustness of fish segmentation. The ECA mechanism can help the model adaptively focus on the important features in the image and reduce the attention to irrelevant information, thereby improving the model’s ability to capture fish features. This improvement can not only improve the accuracy of segmentation but also enhance the ability of the model to adapt to various fish forms and background changes, so that it can still stably and effectively segment fish in complex environments. Therefore, applying the ECA mechanism to the DeepLabV3+ model can deliver significant performance improvement and more reliable results for fish segmentation tasks. Figure 4 shows the network structure diagram with the ECA mechanism added.

### 3.4. FAIvar Fish Feeding Behavior Recognition Module

**Figure 5:**
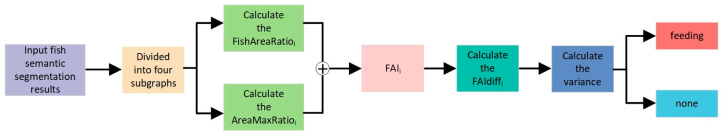
Flowchart of FAIvar module.

The proposed FAIvar model first uses a semantic segmentation model (ECA-Deeplabv3+) to perform high-precision image segmentation of fish in the water. This step consists of classifying each pixel as fish or background, thus forming an accurate fish segmentation image. Then, the segmented image is input into the FAIvar fish feeding behavior recognition algorithm proposed in this paper, and the overall flow chart of the algorithm is shown in Figure 5.

**Figure 6:**
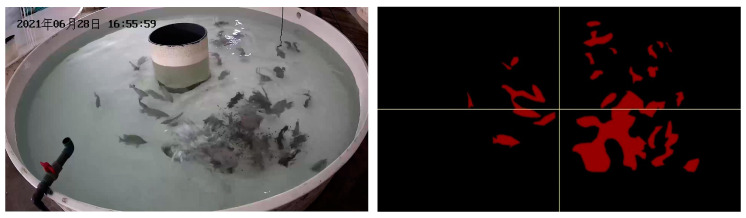
An example of dividing the fish semantic segmentation result illustration into four equally sized regions.

**Figure 7:**
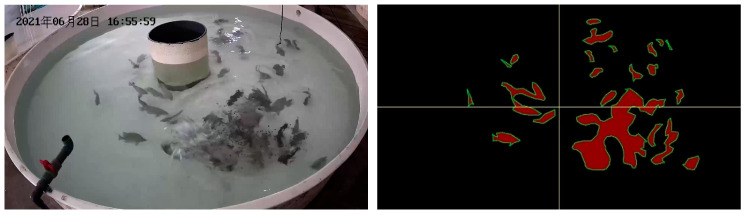
Example illustration of calculating fish area percentage.

**Figure 8:**
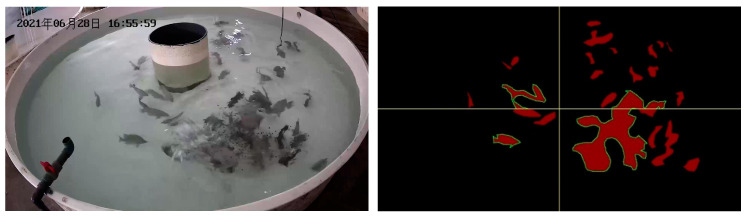
Example illustration of calculating maximum fish area percentage.

Firstly, the FAIvar feeding behavior recognition model equally divides the segmented image into two halves according to the horizontal and vertical directions, and then obtains four equally large sub-images to observe the situation of different areas of the water in more detail. An example figure is shown in Figure 6. For the convenience of subsequent analysis, the upper left label in the picture is 1, the upper right label is 2, the lower left label is 3, and the lower right label is 4. Then, each sub-image is analyzed. Firstly, the percentage of fish area in the sub-image is calculated, which can reflect the approximate number of fish in the sub-image area of the current frame and analyze the distribution characteristics of fish in the water. A sample figure is shown in Figure 7, and the formula is shown in (1). Then, the maximum area percentage of all segmented regions in the sub-image is calculated, and this percentage can clearly show the aggregation of fish. A sample figure is shown in Figure 8, and its formula is shown in (2).

(1) $${FishAreaRatio}_{i}=\frac{A_{{fish}_{i}}}{A_{{total}_{i}}}\times 100\%$$

(2) $${AreaMaxRatio}_{i}=\frac{A_{{max}_{i}}}{A_{{total}_{i}}}\times 100\%$$

Calculate the percentage of the maximum fish area and add it to the percentage of fish area to obtain the Fish Feeding Activity Index. This value not only reflects the distribution of fish schools in each area but also reveals their aggregation characteristics. Its formula is shown in (3).

(3) $${{Feeding\;Activity\;Index}_{i}=FishAreaRatio}_{i}+{FishAreaRatio}_{i}$$

In the following, we abbreviate the Index of Fish feeding behavior as FAI. Through the calculation, we can obtain the FAI value of area 1 to 4 in the current frame. However, this index of fish feeding activity only reflects the distribution and aggregation of fish in the current frame. In order to obtain a more comprehensive understanding of fish feeding, we introduce the concept of time series.

Our method involves comparing the Feeding Activity Index (FAI) of four regions in the current frame with the FAI values of the same regions from the previous 20 frames to observe changes in fish behavior over time. Specifically, we calculated the difference between the FAI value of the current frame and the FAI value of the same region 20 frames earlier to obtain four sets of FAIdiff values. Then, we used the variance to measure the degree of dispersion of the four sets of data.

(4) $${FAIdiff}_{i}={FAI}_{t-20\left(i\right)}-{FAI}_{t(i)}$$

Over a period of time, if the fish are in a non-feeding state, their movement is usually more gradual, which means that the FAIdiff values of the four regions vary less, and therefore the variance obtained is smaller. However, when fish are feeding, they usually swim rapidly to one of the four regions, resulting in a significant increase in the FAI value in that region, while the FAI values in the other regions decrease. In this case, the FAIdiff values of the four regions show a large dispersion, thus resulting in a large variance.

(5) $$\bar{AvgFAIdiff}=\frac{1}{4}\sum_{i=1}^{4}{FAIdiff}_{i}$$

In short, when fish are feeding, their FAIdiff values in the four regions tend to show a large variation, which is reflected in the results with a large variance.

(6) $$Var(FAI)=\frac{1}{4}\sum_{i=1}^{4}{\left({FAIdiff}_{i}-\bar{AvgFAIdiff}\right)}^{2}$$

When fish are feeding, their feeding behavior may vary from region to region. Specifically, when fish feed in an area, the index of feeding behavior in that area increases, while the index of feeding behavior in other areas decreases. We can measure the dispersion of these indices by the variance, and thus quantify the feeding behavior of fish.

To assess the feeding behavior of fish, we introduce a new metric called FAIvar. We set a threshold: when the FAIvar is greater than the set threshold, we consider the fish to be engaged in feeding behavior; otherwise, we considered it as a non-feeding behavior, and this indicator allows us to observe the feeding of the fish more accurately. In this way, we can obtain a more accurate picture of the feeding behavior outcome of fish.

## 4. Experimental Experiment

### 4.1. Datasets and Implementation Details

Fish feeding behavior recognition is a key step in intelligent feeding. To achieve this goal, we use two main fish feeding behavior datasets to validate the proposed method: one is the fish semantic segmentation dataset (DLOUSegDataset), and the other is the aquatic fish feeding behavior video taken by Cui [30].

**Figure 9:**
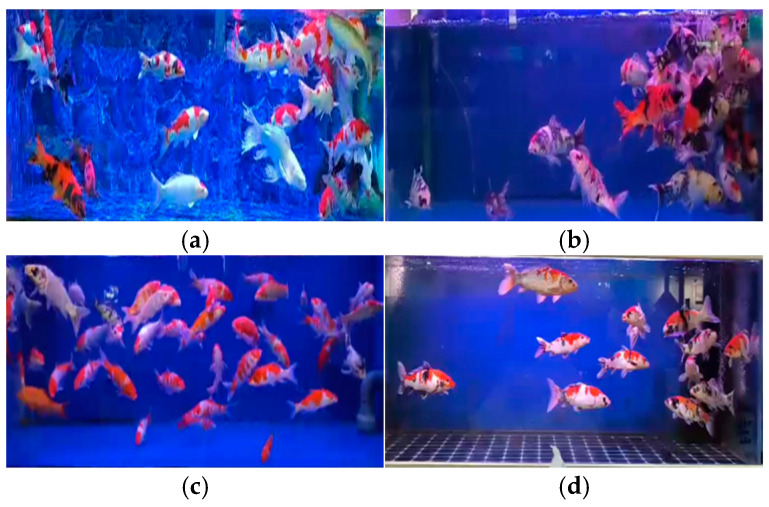
Example of DLOUSegDataset dataset. (**a**,**b**) represent examples of fish feeding behavior, while (**c**,**d**) represent examples of non-feeding behavior.

Our fish semantic segmentation dataset (DLOUSegDataset) contains 40 complete videos of fish feeding behavior, which are filmed from the side of the fish, and the length of time ranges from 2 min to 10 min. We annotated 900 randomly selected frames from the video data, which contain aggregated pictures when fish are feeding and scattered pictures when fish are not feeding. An example of the DLOUSegdataset dataset is shown in Figure 9.

**Figure 10:**
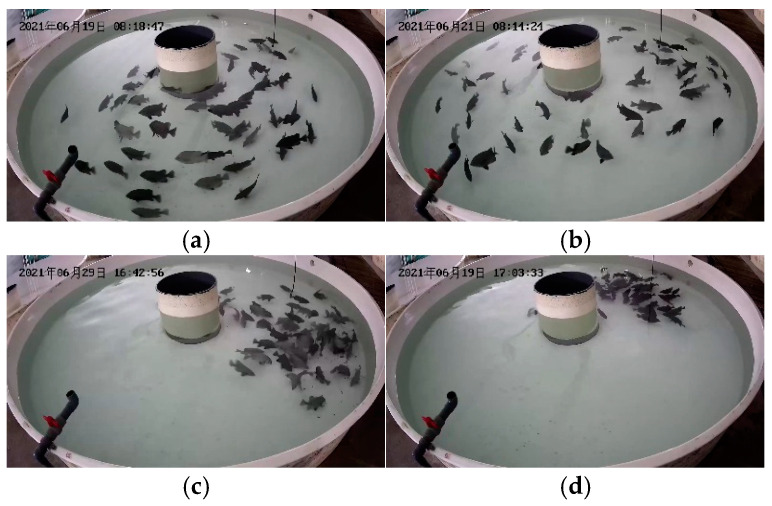
Dataset of fish feeding behavior taken by Cui et al. (**a**,**b**) represent examples of fish non-feeding behavior, while (**c**,**d**) represent examples of feeding behavior.

The other is a video of aquatic fish feeding behavior taken by Cui. The fish in this dataset are raised in a circulating pool with a diameter of three meters and a depth of 0.75 meters. The number of fish in this dataset is 60, including fish feeding and non-feeding behavior. An example of this is shown in Figure 10. We sampled 500 frames in Cui’s dataset as labeled data. This dataset was collected from the water surface, which is complementary to our dataset and enhances the reliability of our method. Again, we used the same annotation strategy to label these data frames.

In the experiments, the combination of these two datasets provided an extensive validation of our approach, allowing us to comprehensively evaluate the performance and generalization ability of the proposed model.

In the process of training and predicting fish feeding behavior models, the quality of data annotation was crucial to the performance of the model. To ensure accurate annotation, the images in the dataset were annotated using LabelMe version 4.5.13. During the annotation process, we annotated the fish in its entirety when it was fully visible. The purpose of this was to provide the model with as accurate and comprehensive information as possible to help it learn the morphological and appearance characteristics of the fish for better recognition and segmentation.

The purpose of labeling the largest bounding box of fish is to take into account the aggregation phenomenon that may occur when fish are feeding. At a time when a school is feeding, fish tend to cluster together, causing their body parts to overlap with each other or be occluded by other fish. This can manifest itself in images where part of the fish body is not fully visible, and only part of the body or just part of the fish can be seen.

Therefore, in order to still provide accurate annotation information in this case, we chose the largest bounding box that labels the overlapping fish. Labeling the largest bounding box ensured that we covered all visible fish parts as much as possible, and could accurately label the overall outline of the school of fish even if part of the body was occluded by other fish or objects. Such a labeling strategy helps to provide more comprehensive and accurate training data, so that the model can recognize and segment fish more robustly in the face of fish aggregation and overlap.

### 4.2. Evaluation Indicators

In order to verify the effectiveness of our example segmentation for fish segmentation, we evaluated the performance of the model from the following two indicators: Mean intersection over Union (mIoU) and Mean Average Precision (mAcc).

Intersection over Union (IoU) is one of the commonly used evaluation metrics in the semantic segmentation module, and is used to measure the degree of overlap between the model predicted segmentation results and the true segmentation. IoU is measured by calculating the ratio between the intersection area and the union area of the predicted segmentation region and the true segmentation region. Specifically, the IoU is calculated using the formula shown in Equation (7).

(7) $$IoU=\frac{{Area}_{Intersection}}{{Area}_{Union}}$$

AreaIntersection represents the intersection area of the predicted segmentation region and the true segmentation region, and AreaUnion represents the union area of the predicted segmentation region and the true segmentation region.

The value of IoU ranges from 0 to 1, and the larger the value of IoU, the higher the degree of overlap between the predicted segmentation result and the true segmentation, that is, the better the segmentation effect of the model. An IoU equal to 1 means that the predicted segmentation is completely consistent with the true segmentation; an IoU equal to 0 means there is no overlap between the two.

Mean Intersection over Union (mIoU) is calculated as the average of IoU (Intersection over Union) and is used to measure the segmentation performance of the model on all classes. The mIoU is calculated by first calculating the IoU of each class, and then averaging the IoU of all classes to obtain the final mIoU.

Specifically, assuming that there are N categories and the IoU of each category i is IoUi, the formula for calculating mIoU is shown in Equation (8):

(8) $$mIoU=\frac{1}{N}\sum_{i=1}^{N}{IoU}_{i}$$

The mIoU values also range from 0 to 1, with higher values indicating better model performance across all classes. mIoU is a comprehensive performance index that can comprehensively evaluate the segmentation accuracy of the model on each category, and it is therefore widely used in the evaluation of semantic segmentation tasks.

mIoU not only considers the segmentation accuracy of each class but also considers the number of pixels in each class, so it can reflect the segmentation ability of the model more comprehensively

Mean Pixel Accuracy (mAcc) is used to measure the average of the classification accuracy of the model over all pixels. Similarly to mIoU, mAcc is an aggregate performance measure, but instead of considering the intersection over union between each class, MACC directly averages the classification accuracy over all pixels.

The mAcc calculation formula is shown in Formula (9).

(9) $$mAcc=\frac{1}{N}\sum_{i=1}^{N}\frac{{TP}_{i}}{{TP}_{i}+{FP}_{i}}$$

mAcc calculates the average of the model’s classification accuracy for each class, so it can give us an idea of the overall classification performance of the model. Unlike Pixel Accuracy, mAcc takes into account the difference in the number of pixels between each class, so it gives a more comprehensive picture of the model’s classification accuracy.

### 4.3. Evaluation Configuration Experimental Environment

Our experiments were conducted on a desktop computer equipped with an Intel Core i7 processor (3.6 GHz), 32 GB of RAM, and an NVIDIA GeForce RTX 3090 graphics card. The operating system is Ubuntu 20.04 LTS, Python 3.8 programming language is used, and PyTorch 1.10.0 is adopted as the deep learning framework. The experimental dataset is from DLOUSegDataset and fish videos captured by Cui. After screening, we divided the 1400 images into a training set, validation set, and test set according to the ratio of 8:1:1. In the experiments, we used the SGD optimizer with a learning rate of 0.01 for model training, batch size of 12, and set 2000 epochs for training. The specific experimental environment is shown in Table 2.

To better adapt to aquaculture environments, we transferred the trained model to the NVIDIA Jetson Nano edge computing board for testing. This board, with its low power consumption and compact design, is highly suitable for deployment in real-world aquaculture settings. By running the model on the Jetson Nano, we obtained favorable results, demonstrating that the hardware can support real-time processing and accurately recognize fish feeding behaviors.

## 5. Experimental Results and Analysis

### 5.1. Semantic Segmentation Results and Analysis

**Figure 11:**
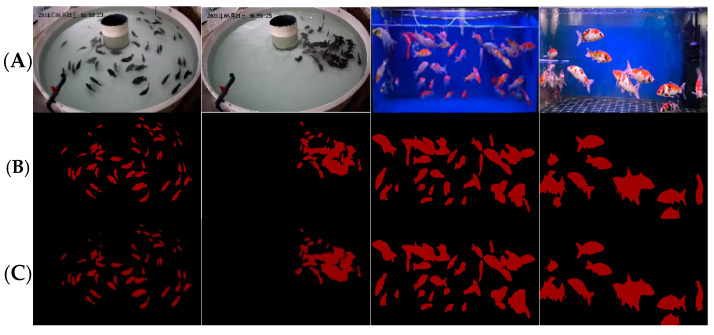
Example of ECA-Deeplabv3+ semantic segmentation results: (**A**) original image, (**B**) annotated picture, (**C**) prediction results.

After experimental verification, we observed that the performance of Deeplabv3+ was significantly improved after adopting the ECA mechanism. As shown in Table 2, compared with the traditional PSPNet, U-Net, and HRNet, ECA-Deeplabv3+ showed significant advantages, achieving 93.61% average IoU and 94.97% average accuracy, which far exceeds other models. The semantic segmentation results of ECA-Deeplabv3+ are shown in Figure 11. These results not only demonstrate the effectiveness of our algorithm in dealing with complex spatio-temporal features and fine classification, but also provide strong support for further research and application in the field of fish feeding behavior recognition.

By accurately improving the fish image segmentation algorithm, our goal is not only to improve the accuracy of segmentation, but more importantly to provide more reliable input for subsequent fish feeding behavior recognition algorithms. Since the feeding behavior recognition algorithm depends on the accurate segmentation of fish images, the accuracy of the segmentation results directly affects the performance of subsequent algorithms. Therefore, we focused on improving the performance of the segmentation model to ensure that the segmentation results can provide accurate and reliable information for the feeding behavior recognition algorithm.

Comparison of experimental results in Table 3.

### 5.2. Feeding Behavior Recognition Results and Analysis

The following are some technical details about the data: Standard overhead LED lighting was used in the experiment to ensure consistent illumination throughout, simulating a typical indoor aquaculture facility. The light intensity ranged from 500 to 700 lux, providing sufficient brightness for clear observation and image capture while avoiding glare. The water transparency was maintained at approximately 40–50 cm, representing clean but realistic aquaculture conditions. The water temperature ranged from 15 to 25 °C. Regular water changes ensured stable water quality, minimizing the interference of suspended particles and other environmental factors. Feeding followed standard aquaculture protocols, with two feedings daily at 8:00 a.m. and 5:00 p.m. These feeding times were chosen to ensure consistent intervals for observing feeding behavior and to allow for sufficient digestion time between feedings, maintaining uniform experimental conditions.

Regarding the FAI (Feeding Activity Index) threshold, its selection is crucial as it directly affects the accuracy of feeding behavior recognition. The threshold was dynamically adjusted based on species-specific feeding patterns observed during experiments. For species exhibiting intense feeding behavior, a higher threshold was set to better identify aggregation, while also reducing misjudgments in ambiguous situations.

In addition, the frame difference can be adjusted to optimize the detection of feeding behavior. Specifically, for species with slower feeding behavior, the frame difference can be increased, allowing the model to analyze the changes in fish aggregation over a longer period. This adjustment enables the method to more precisely capture the dynamics of fish feeding behavior.

By combining these two parameters—the FAI threshold and the frame difference—our method provides a robust and adaptable approach to accurately detect fish feeding behavior under varying conditions.

**Figure 12:**
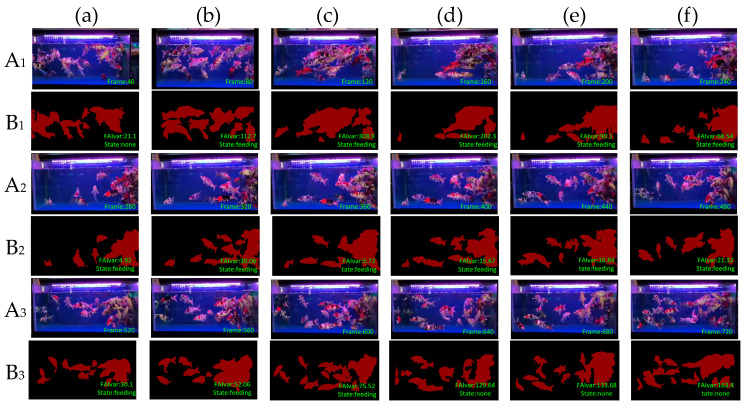
Example of feeding behavior recognition results on Dlousegdataset. (**A**) Input image, (**B**) prediction. To provide a clearer explanation of this section, we have added subscript numbers and lowercase letters. The video frames are arranged in chronological order from smallest to largest, for example, **A_1_**(**a**)–**A_3_**(**f**) represent a complete video sequence.

**Figure 13:**
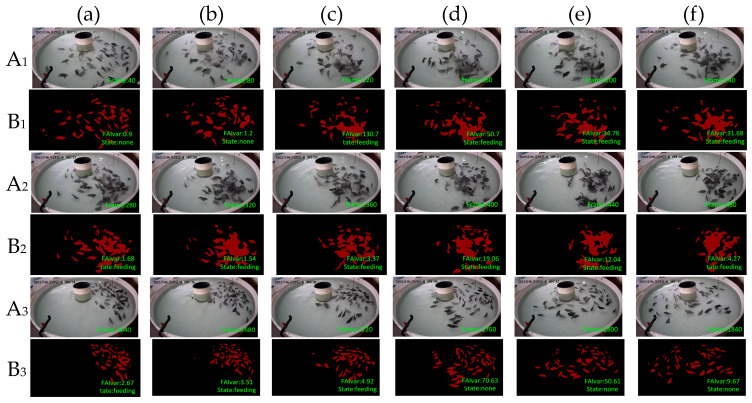
Example results of feeding behavior recognition on Cui dataset. (**A**) Input image, (**B**) prediction. To provide a clearer explanation of this section, we have added subscript numbers and lowercase letters. The video frames are arranged in chronological order from smallest to largest, for example, **A_1_**(**a**)–**A_3_**(**f**) represent a complete video sequence.

To verify the effectiveness of our feeding behavior recognition algorithm, we tested it on DlouSegDataset and Cui’s datasets. The experimental results show that our fish semantic segmentation module can efficiently and accurately distinguish the feeding behavior of fish from other behaviors, and an example is shown in Figure 12 and Figure 13.

Depending on the species of fish, different thresholds can be set to determine whether a fish is feeding. The threshold for determining the feeding behavior of fish in DLOUSegDataset is set to 100. It can be seen from B_1_(a) in Figure 12 that the fish are swimming without feeding behavior at this time. It can be seen from Figure 12B_1_(b)–B_1_(d) that the bait was being cast at this time, and the fish were rapidly gathering to the right area, FAIvar > 100, which indicates that the fish were feeding. From Figure 12B_1_(e) to B_3_(c), it can be seen that the school was feeding, and there was no obvious swimming in the area. FAIvar < 100, so it was judged that the school was feeding. From Figure 12B_3_(d) to B_3_(f), it can be seen that the fish were feeding and started to swim to other areas. FAIvar > 100, it is judged that the fish were not feeding.

In order to verify the robustness of our experiments, we also verified our algorithm on Cui’s dataset. Because the fish species in the two datasets are different, the feeding behavior is also different. The fish in Cui’s dataset are small and the feeding behavior is intense, so the threshold on this dataset was set to 50. From Figure 13B_1_(a)–B_1_(b), we can see that the fish were swimming, and the FAIvar was <50 at this time, which means the fish were not feeding. In frame Figure 13B_1_(c)–B_1_(d), bait casting is started, and the fish quickly swim to the bait casting area. At this time, FAIvar > 50, the fish were judged as showing feeding behavior. According to Figure 13B_1_(e)–B_3_(c), the fish were feeding without obvious swimming behavior, and FAIvar < 50, so the fish were judged as showing feeding behavior. From Figure 13B_3_(d) to B_3_(e), the fish feeding was completed and the fish started to swim. At this time, the FAIvar > 50, the fish showed non-feeding behavior.

In certain ambiguous feeding scenarios, where the fish aggregation behavior may not clearly indicate feeding activity, the model can be enhanced by utilizing a trial feeding approach. During the trial feeding phase, the model observes the fish’s response to the initial feed to assess whether they exhibit clear feeding behavior. If fish show an evident feeding response, the system confirms the feeding activity and proceeds with the feeding plan. If the response is minimal or absent, it indicates that the aggregation behavior may not be related to feeding, and the model will not recognize it as such.

We randomly selected 100 fish feeding videos in the dataset, and the accuracy rate reached 90%, thus demonstrating the effectiveness and reliability of our algorithm. Our method not only improves the accuracy of fish semantic segmentation, but also provides strong support for the effectiveness and practicability of feeding behavior recognition algorithms. This result is expected to provide important technical support for the in-depth understanding of fish feeding behavior and its ecological significance, and to provide new ideas and methods for the protection and management of aquatic ecosystems.

### 5.3. Limitation Analysis

Although our model addresses some of the challenges in feeding behavior recognition in aquaculture, there are still limitations that prevent it from perfectly adapting to and accurately recognizing feeding behaviors in all environments. Specifically, the model’s performance may vary across different environments, especially in the following scenarios:

1. Issues in High-Density Aquaculture Tanks: In high-density aquaculture tanks, the aggregation behavior of fish may not be apparent, or there may be no clear aggregation or dispersal process at all. In such environments, the density of the fish is higher, and the distance between individuals is smaller, making it difficult to identify aggregation features through visual information. Furthermore, the feeding actions and behavioral patterns of fish may be more tightly grouped and harder to distinguish, making it challenging for vision-based segmentation methods to effectively extract the contours of individual fish, which in turn affects the model’s ability to accurately identify behaviors.
2. Complex Background and Water Quality Issues: In aquaculture environments with poor water quality or weak lighting, image quality can be significantly compromised, leading to increased noise in the visual data. Factors such as suspended particles in the water and light refraction may blur the contours of the fish, affecting the accuracy of the segmentation algorithm. Although our model mitigates these issues to some extent through dilated convolutions and multi-scale feature extraction, extreme environmental conditions may still result in visual information that cannot effectively capture feeding behaviors.

Therefore, although our model has demonstrated high recognition accuracy in standardized aquaculture environments, its performance is still limited under certain special or extreme conditions. Future research will need to further optimize the model to enhance its adaptability to these complex scenarios. To compensate for the limitations of visual data, we could also consider integrating acoustic information, sensor data, or multimodal learning methods to improve the model’s robustness and accuracy in various environments.

### 5.4. Potential Impacts on Animal Welfare

In intensive aquaculture environments, the welfare of fish is a critical concern, especially with the increasing use of continuous monitoring technologies. While these technologies improve management, they can also cause stress and disrupt natural behaviors in fish. Frequent monitoring, such as the use of underwater cameras or sensors, can disturb fish, leading to stress and changes in their feeding, schooling, and resting behaviors. This stress may negatively affect their health, growth, and overall well-being. To minimize the negative impact on fish, it is important to use non-invasive monitoring methods, such as underwater cameras and sensors, that do not physically interfere with the fish. Monitoring frequency should be regulated to avoid overexposure and reduce behavioral disruption. Additionally, long-term monitoring must be carefully considered to prevent chronic stress, which could lead to health issues such as reduced growth or immune dysfunction.

## 6. Conclusions

In order to provide powerful guidance and decision support for the bait casting machine, this paper proposes a fish feeding behavior recognition method based on semantic segmentation. In this method, the fish target is first segmented, and then the fish feeding behavior model is determined by the aggregation of fish feeding. By adding the ECA mechanism to the semantic segmentation module, the segmentation accuracy of fish targets is further improved. By importing the results of fish semantic segmentation into the FAIvar fish feeding behavior recognition module in real time, the feeding status of fish can be obtained. This method can not only help to monitor the feeding behavior of fish in the water, but also provides detailed information about the activity of different areas, which can help farmers better understand the dynamic changes in fish behavior, and optimize aquaculture strategies and management decisions, thereby improving the production efficiency and economic benefits of the aquaculture industry. Therefore, this research method has important practical significance and application value, and it provides effective technical support for intelligent management in the field of aquaculture.

In the future, we plan to incorporate acoustic information from the fish feeding process, combining visual and sound data for a more comprehensive analysis of feeding behavior. Acoustic data have high transmission stability in underwater environments, which can help mitigate some limitations of visual data, such as issues caused by lighting variations, occlusions, and water quality interference. By leveraging the complementary strengths of both data types, we aim to enhance the accuracy and robustness of feeding behavior detection, ultimately providing more reliable data support for intelligent aquaculture management.

## Figures and Tables

**Table 1:** A comprehensive overview of fish behavioral recognition.

References	Method	Number	Shortcoming
Atoum et al. [8]	Machine learning	Fish school	Low accuracy
Zhou et al. [9]	Machine learning	Fish school	Overlapping impact
Zhu et al. [19]	Convolution	Fish school	No consideration of time
Ubina et al. [22]	3D convolution	Fish school	Poor real-time performance
Yang et al. [23]	Segmentation	Fish school	No consideration of time

**Table 2:** Experimental environment.

Configuration	Parameter
Operating system	Ubuntu 20.04 LTS
graphics card	NVIDIA GeForce RTX 3090
CUDA version	11.1
Python version	3.8
Pytorch version	1.10.0
Development environment	Pycharm2022

**Table 3:** Algorithm performance comparison.

Algorithm	mIoU/%	mAcc/%
PSPNet	74.99	75.87
U-Net	83.75	85.75
HRNet	84.86	85.95
Deeolabv3+	90.51	92.67
ECA-Deeplabv3+	93.61	94.97

## Data Availability

The data presented in this study are available on request from the corresponding author.
